# Preanalytical blood sample workup for cell‐free DNA analysis using Droplet Digital PCR for future molecular cancer diagnostics

**DOI:** 10.1002/cam4.1184

**Published:** 2017-09-21

**Authors:** Joost H. van Ginkel, Daan A. van den Broek, Joyce van Kuik, Dorothé Linders, Roel de Weger, Stefan M. Willems, Manon M. H. Huibers

**Affiliations:** ^1^ Department of Pathology University Medical Center Utrecht The Netherlands; ^2^ Department of Clinical Chemistry Netherlands Cancer Institute Amsterdam The Netherlands

**Keywords:** Cell‐free DNA, droplet digital PCR, molecular cancer diagnostics, preanalytical workup

## Abstract

In current molecular cancer diagnostics, using blood samples of cancer patients for the detection of genetic alterations in plasma (cell‐free) circulating tumor DNA (ctDNA) is an emerging practice. Since ctDNA levels in blood are low, highly sensitive Droplet Digital PCR (ddPCR) can be used for detecting rare mutational targets. In order to perform ddPCR on blood samples, a standardized procedure for processing and analyzing blood samples is necessary to facilitate implementation into clinical practice. Therefore, we assessed the technical sample workup procedure for ddPCR on blood plasma samples. Blood samples from healthy individuals, as well as lung cancer patients were analyzed. We compared different methods and protocols for sample collection, storage, centrifugation, isolation, and quantification. Cell‐free DNA (cfDNA) concentrations of several wild‐type targets and *BRAF* and *EGFR*‐mutant ctDNA concentrations quantified by ddPCR were primary outcome measurements. Highest cfDNA concentrations were measured in blood collected in serum tubes. No significant differences in cfDNA concentrations were detected between various time points of up to 24 h until centrifugation. Highest cfDNA concentrations were detected after DNA isolation with the Quick cfDNA Serum & Plasma Kit, while plasma isolation using the QIAamp Circulating Nucleic Acid Kit yielded the most consistent results. DdPCR results on cfDNA are highly dependent on multiple factors during preanalytical sample workup, which need to be addressed during the development of this diagnostic tool for cancer diagnostics in the future.

## Introduction

Current cancer diagnostics is often performed on molecular pathology findings from biopsy material. This is an invasive technique and not always possible to perform. A less invasive method for molecular diagnostics is the use of blood (i.e., “liquid biopsy”). Blood samples are easy to obtain and contain cell‐free DNA (cfDNA) including circulating tumor DNA (ctDNA). These DNA fragments carry patient‐specific genetic targets, and can be used as a diagnostic, prognostic or predictive biomarker. This enables new strategies for personalized cancer medicine and other clinical fields like prenatal testing, transplantation medicine, and traumatology 1. Highly sensitive droplet digital PCR (ddPCR) is capable of detecting these, often rare, targets 2, 3. For optimal workflow, a standardized procedure is required for processing and cfDNA analysis 4, 5. To date, different pre‐ and postanalytical approaches have been studied substantially for the development of molecular cancer diagnostics on liquid biopsies using quantitative PCR (qPCR) 6, 7, 8, 9. However, no standardized approach exists for the use of liquid biopsy in conjunction with ddPCR, which has only recently been introduced into molecular diagnostics and clinical research. This could explain inconsistencies in blood sample workup using ddPCR on liquid biopsies.

We aim to generate a more standardized procedure for molecular testing on liquid biopsy using ddPCR. Here, we describe options of preanalytical methods, in which we evaluated blood collection, storage of samples, centrifugation protocols, and isolation and quantification methods of cfDNA.

## Methods

### Subjects and samples

Blood samples of 46 different healthy individuals and 18 lung cancer patients were used for analysis, as well as three pools each consisting of blood samples from 60 patients with various cancer types. Lung cancer patients were all NSCLC patients with progressive disease under erlotinib or gefitinib. Both primary driver mutation (*EFGR E746‐A750del*,* G719S*, or *L858R*) and resistance mutation (*T790M*) were analyzed during treatment. The healthy blood samples were retrieved from the anonymous blood donation biobank in the Utrecht University Medical Center (center A) and the Netherlands Cancer Institute (center B). Blood samples from cancer patients were all leftover material from the Netherlands Cancer Institute. According to Dutch national ethical guidelines, no ethical approval to use leftover material for scientific purposes is required, as the use of anonymous leftover material and clinical data for scientific purposes is part of the treatment agreement with patients 10.

### Blood sample collection, room temperature storage, and centrifugation

**Figure 1:**
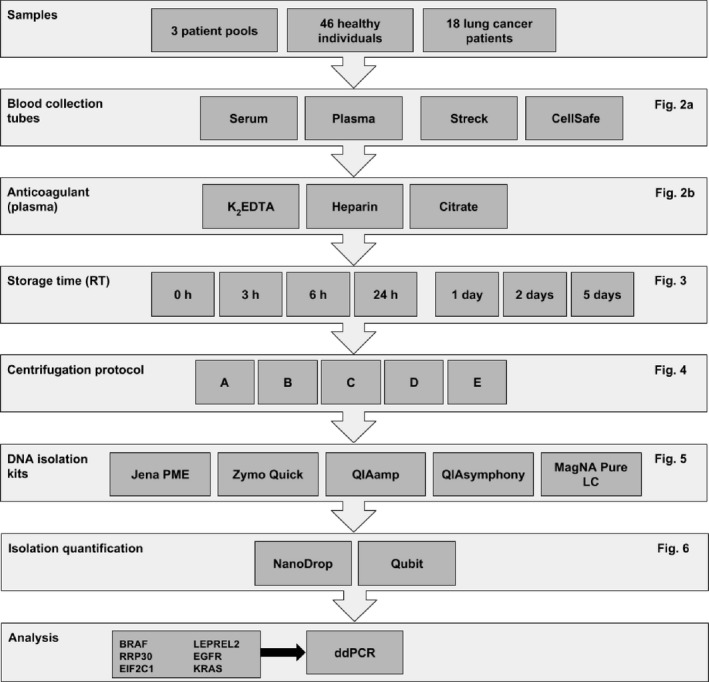
Summary of materials and methods used during various experiments. Please note: this is a schematic overview of the experimental workflow. No exact experiments are depicted.

To test how blood sample collection, storage time, and centrifugation affect cfDNA quality and quantity, different materials and protocols were compared (Fig. 1). All patient samples and methods used for analysis are summarized in Table S1. Whole blood was collected in CellSave Preservative Tubes (Janssen Diagnostics, Raritan, NJ, USA), Cell‐Free DNA blood collection tubes (BCT) (Streck Inc, La Vista, NE, USA), *K*_2_/*K*_3_ ethylenediaminetetraacetic acid (EDTA), heparin, silicone coated (for serum separation) and citrate (BD Vacutainer, Franklin Lakes, NJ, USA) BCT. Samples were stored for 0 (*T*_1_), 3 (*T*_2_), 6 (*T*_3_), and 24 (*T*_4_) h at room temperature (RT) until centrifugation. Blood samples collected in Streck and CellSafe BCTs were stored for 24 (*T*_4_) h (P13–P14), 2 days (*T*_5_) (P15–P16), and 5 days (*T*_6_) (P17–P18). Centrifugation force could also affect levels of cfDNA as lysis of white blood cells in blood samples increase with increasing centrifugation force, which in turn increases background DNA concentration. Furthermore, less purified blood samples contain more cellular debris (e.g., proteins, nucleic acids), possibly leading to suboptimal DNA isolation and higher PCR interference. We performed and compared a one‐step (slow‐speed) and four different two‐step (slow‐ and high‐speed) centrifugation methods using the following centrifugation protocols of which parameters were based on standard clinical practice in both centers: (A) prefreeze centrifugation for 10 min at 800*g* (Rotina 380, Hettich, Germany), (B) prefreeze centrifugation for 10 min at 800*g* and followed by microcentrifugation for 1 min at 11,000*g* (5424 Microcentrifuge, Eppendorf, Germany), (C) prefreeze centrifugation for 10 minutes at 800*g* prefreeze followed by post‐thaw microcentrifugation of 1 min at 11,000*g*. Additionally, slightly modified protocols B (20 min at 380*g* and 10 min at 20,000*g* prefreeze) and C (20 min at 380*g* prefreeze and 10 min at 20,000*g* post‐thaw) were handled, named protocol D and E, respectively (Table 1). After each slow‐speed centrifugation step, supernatant plasma was carefully removed from the tube and transferred into 1 mL aliquots. After each high‐speed centrifugation step, supernatant plasma was transferred to a new 1.5 mL tube. All plasma samples were stored at −20°C.

**Table 1:** Centrifugation protocols

Protocol	1st centrifugation			2nd centrifugation		
	Timing	Force (g)	Time (min)	Timing	Force (g)	Time (min)
A	Prefreeze	800	10	Not performed	N/A	N/A
B	Prefreeze	800	10	Prefreeze	11,000	1
C	Prefreeze	800	10	Post‐thaw	11,000	1
D	Prefreeze	380	20	Prefreeze	20,000	10
E	Prefreeze	380	20	Post‐thaw	20,000	10

### DNA isolation methods

For comparison of DNA isolation methods, the following isolation kits were used: Jena PME free‐circulating DNA extraction kit (Analytik Jena, Germany), QIAamp Circulating NA Kit (Qiagen, Hilden, Germany), QIAsymphony Circulating NA kit (Qiagen, Hilden, Germany), MagNAPure LC Total Nucleic Acid Isolation Large Volume kit (Roche Life Science, Basel, Switzerland), and Zymo Quick cfDNA serum & plasma kit (Zymo Research, Irvine, CA, USA). Kit specifications are shown in Table 2. All experiments were performed using 1–2 mL of plasma according to the manufacturer's protocol. Since the MagNA Pure kit allows a maximum plasma input of 1 mL, all other isolations methods were performed with 1 mL plasma to gain comparable results. DNA isolated from healthy donor plasma was eluted in 50 *μ*L elution buffer provided by the kit manufacturers. DNA isolated from plasma from lung cancer patients was eluted in 60 *μ*L elution buffer.

**Table 2:** Cell‐free DNA isolation kit specifications

cfDNA isolation kit	Manufacturer	Plasma input (mL)	Technique	Carrier RNA
PME free‐circulating DNA extraction kit	AnalytikJena	1–5	Spin‐based	Optional
QIAamp circulating NA Kit 50	Qiagen	3–5	Vacuum‐based	Yes
QIAsymphony circulating DNA Kit	Qiagen	4	Automated	Optional
MagNA pure LC DNA isolation kit – large volume	Roche	1	Beads‐assisted	No
Quick cfDNA serum & plasma kit	Zymo research	10	Spin‐based	No

### DNA isolation quantification

Quantification of nucleic acids within eluates after DNA isolation was performed using NanoDrop 2000 spectrophotometry (Thermo Fisher Scientific, Waltham, MA, USA) and Qubit 3.0 fluorometry (Thermo Fisher Scientific) according to manufacturer's instructions (using 1 *μ*L samples of DNA eluates). Both DNA quantification methods were analyzed by correlating measured values with ddPCR results of identical samples.

### ddPCR analysis

Droplet digital PCR was performed using several assays containing primers and probes targeting wild‐type *BRAF* (1), *RPP30* (2), *EIF2C1* (3), a single‐nucleotide polymorphism (SNP) variant of *LEPREL2* (4), 6 mutant *EGFR* (5–9), and 8 mutant *KRAS* (10 and 11 [G12/G13 screening multiplex assay]). DNA templates used during PCR are shown in Table S2 following MIQE guidelines for digital PCR 11. Initial PCR mix volume consisted of 12 *μ*L mastermix (11 *μ*L supermix for probes [no uDTP] and 1 or 2 *μ*L of wild‐type assay), and 9 or 10 *μ*L of DNA depending on the amount of assay used. Within the no template control (NTC), DNA was substituted for purified H_2_O (MilliQ, Billerica, MA, USA). All samples were analyzed in duplicate. PCR settings were based on a manually performed temperature gradient or validation data from Bio‐Rad if available. Sample analysis of each experiment was performed using QuantaSoft v1.7.4.0917 software (Bio‐Rad Laboratories, Hercules, CA, USA). Positive droplet concentrations in all samples were determined using manually placed fluorescence thresholds based on negative clusters as detected in the corresponding NTCs. Target DNA concentration (copies/*μ*L) and absolute droplet counts within single samples were used as quantitative outcome measurement, while positive‐to‐total droplet ratios were calculated in order to compare efficiency of different isolation kits.

### Statistical analysis

Paired differences in cfDNA yield were assessed by the Wilcoxon signed‐rank test or Friedman test with Dunn's correction in case of multiple intraindividual comparisons. Linear regression analysis was performed to calculate *R* ^2^ of DNA quantification measurement methods compared to ddPCR results. Statistical analysis was performed using GraphPad Prism software package version 6.02 (GraphPad Software, San Diego, CA, USA). Data are presented as medians with interquartile range (mdn, *q*1–*q*3), or as means with standard deviation (mn±sd). For all comparisons, a value of *P *<* *0.05 was considered to be significant (two‐tailed).

## Results

### Blood collection

**Figure 2:**
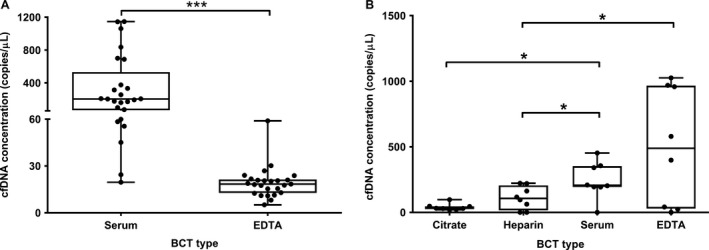
Comparison of cfDNA concentrations in paired blood samples in four different BCTs. All samples originated from healthy controls collected. In all experiments assay, 1 was used during ddPCR. The boxplots indicate cfDNA concentrations on the *y*‐axis, comparing serum with EDTA BCTs from 25 healthy controls on the *x*‐axis (A), and citrate, heparin, serum, and EDTA BCTs from eight other healthy controls (B). The crossing lines indicate medians, the upper and lower limits of the boxes indicate interquartile ranges (25th/75th percentiles), and whiskers represent minima and maxima. *P <* *0.05,***P <* *0.001.

PCR results of blood plasma and serum samples from 10 healthy blood donors (D1–D10) were compared using the MagNA pure kit and another 15 healthy blood donors (D11–D25) were compared using the QIAamp kit isolation method. In all 25 cases, cfDNA concentrations were significantly highest in serum samples compared to paired EDTA samples (204.0 [67.7–532.0] vs. 18.4 [12.7–21.4], *P *<* *0.001) (Fig. 2A). In a second experiment, four different BCTs (EDTA, heparin, serum and citrate) were compared in eight different healthy blood donors (D26–D33). In all cases, the Zymo kit was used for cfDNA isolation between *T*_1_ and *T*_2_. Median cfDNA concentrations (copies/*μ*L) were significantly higher in serum samples compared to paired citrate samples (206.0 [193.5–352.3] vs. 30.8 [24.2–46.4], *P < *0.05) and heparin samples (206.0 [193.5–352.3] vs. 106.5 [15.7–205.8], *P *<* *0.05). Furthermore, significantly higher cfDNA concentrations were found in EDTA samples compared to paired heparin samples (488.5 [28.5–966.3] vs. 106.5 [15.7–20.5], *P *<* *0.05) (Fig. 2B and Figure S1).

### Blood storage time until centrifugation

**Figure 3:**
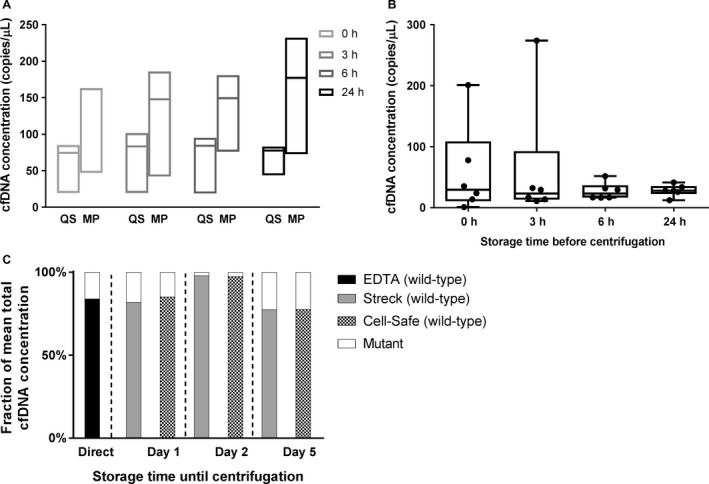
Influence of storage time on cfDNA concentrations until centrifugation. Time points *T*_1_–*T*_6_ are depicted on the *x*‐axes. Median cfDNA concentrations were depicted on the y‐axes for average yields of pooled EDTA samples after analysis using assay 1 (A), and paired EDTA samples from six healthy individuals after using assay 2 (B). In six blood samples collected in Streck and CellSave BCTs using assay 6, 7, 10–12 (C). At each consecutive time point, mean mutant and wild‐type cfDNA concentrations from samples of two other individuals were compared with the mean cfDNA concentration of the matching EDTA samples, as depicted by mutant/wild‐type fractions (*y*‐axis). QS QIAsymphony, MP MagnaPure.

Average total cfDNA concentrations of the three blood sample pools were evaluated for storage time until centrifugation at consecutive time points *T*_1_–*T*_4._ DNA was isolated using MagNA Pure and QIAsymphony kits. Medians of pooled averages ranged from 74.4 to 84.1 copies/*μ*L using QIAsymphony, compared to 147.8–177.1 copies/*μ*L using MagNA Pure (Fig. 3A). Additionally, EDTA samples from six individual subjects (D34–D39) were stored at RT and centrifuged following protocol A at consecutive time points *T*_1_–*T*_4_. DNA was isolated using the Zymo Quick kit. Median cfDNA concentrations did not show any significant differences (*P *=* *0.910) between time points *T*_1_ and *T*_4_ using paired analysis (Fig. 3B and Fig. S2). We also tested cfDNA stability in Streck and CellSave BCTs by comparing mean mutant fractions of cfDNA concentrations in blood samples from two lung cancer patients per time point (*T*4: P13–P14; *T*5: P15–P16; *T*6: P17–P18) after centrifugation using protocol D (Fig. 3C). For directly isolated EDTA samples (*n* = 6), the mutant fraction of mean total cfDNA concentration was 16.0%, compared to mutant fractions of 18.0% and 14.8% at *T*_4_ (*n* = 2), 2.1% and 2.6% at *T*_5_ (*n* = 2), and 22.5% and 22.3% at *T*_6_ (*n* = 2) in Streck and CellSave samples, respectively.

### Centrifugation protocol

**Figure 4:**
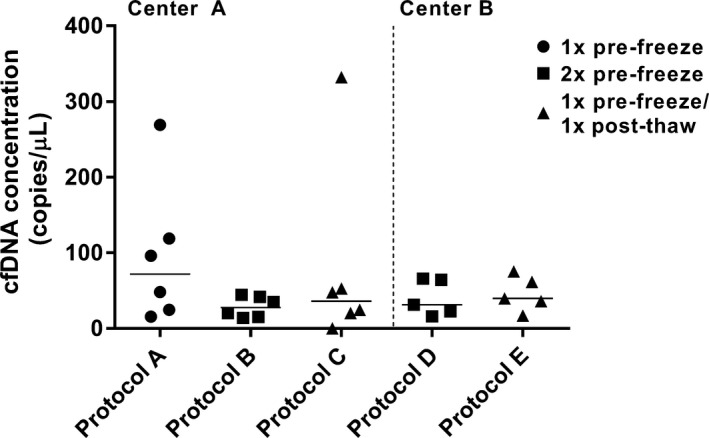
Comparisons of centrifugation protocols A–C, and D and E. Comparisons were performed separately in centers A and B using assay 2 and 5, respectively. Absolute cfDNA concentrations (*y*‐axis) detected in individuals are depicted for each protocol.

Centrifugation protocols A, B, and C were performed at *T*_2_ (3 h) and compared using EDTA plasma from healthy individuals D37–D42, while modified centrifugation protocols D and E were performed on patient samples P1–P5. The median cfDNA concentration detected after centrifugation using protocol A was 72.0 [22.3–156.5] copies/*μ*L, compared to 27.7 [15.0–42.3] copies/*μ*L using protocol B and 36.2 (15.3–122.6) copies/*μ*L using protocol C. The median cfDNA concentration detected after using protocol D was 31.5 [19.2–65.0] copies/*μ*L, compared to 39.8 (26.4–68.5) copies/*μ*L for protocol E (Fig. 4 and Fig. S3).

### Isolation method

**Figure 5:**
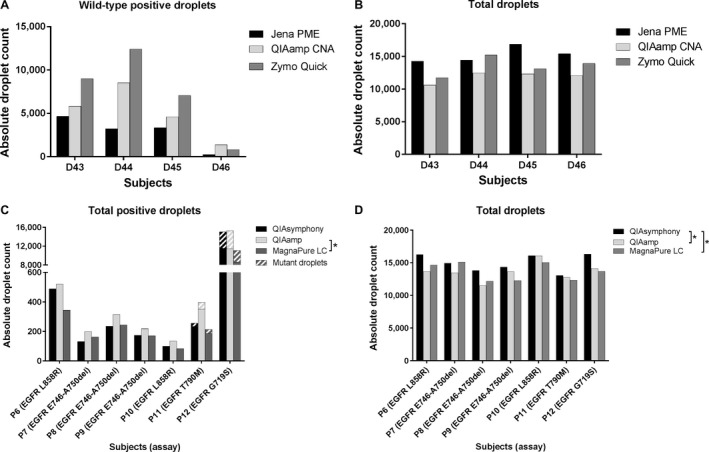
Isolation methods in healthy individuals and cancer patients. Absolute droplets counts are shown on the *y*‐axes. For healthy individuals, wild‐type (A) and total‐positive droplet (B) yield using assay one were depicted. For cancer patients, the sum of mutant and wild‐type positive droplets (C), as well as total droplet yields (D) where depicted using assay 6–9 (*P <* *0.05).

Isolation kits were tested on EDTA samples from D43 to D46, using centrifugation protocol A at *T*_2_ (3 h). Droplet readout of samples (*n* = 4) revealed a mean amount of positive droplets of 2875 ± 1864 and total droplets of 15,261 ± 1196 with a Jena PME kit , a mean positive droplets of 5086 ± 2966 and total droplets of 11,869 ± 861 with a QIAamp kit, and a mean positive droplets of 7339 ± 4867 and mean total droplets of 13,511 ± 1460 with a Zymo Quick kit (Fig. 5A and B). This resulted in positive‐to‐total droplets ratios of 0.19, 0.43, and 0.54, respectively. Identical findings were detected using assay 4 (Fig. S4). In patient samples P6–*P*12, centrifuged using protocol B at *T*_2_, the median total‐positive droplets detected after DNA isolation with the QIAamp kit was significantly higher compared to that detected after isolation with the MagNA Pure kit (316 [199–521] vs. 213 [162–344], *P *<* *0.05). No significant differences were found between median positive droplets by QIAsymphony (236 [132–489]) and the other two kits. Mutant positive droplets were only detected in *P*9, 11, and 12, but did not differ significantly (Fig. 5C). The QIAsymphony kit significantly yielded the highest amount of total droplets compared to both QIAamp (14,942 [13,825–16,246] vs. 13,645 [12,752–14,132], *P *<* *0.05) and MagNA Pure (14,942 [13,825–16,246] vs. 13,705 [12,260–15,028], *P *<* *0.05) kits. No significant differences were found between QIAamp and MagNA Pure kits (Fig. 5D).

### DNA quantification prior to ddPCR

**Figure 6:**
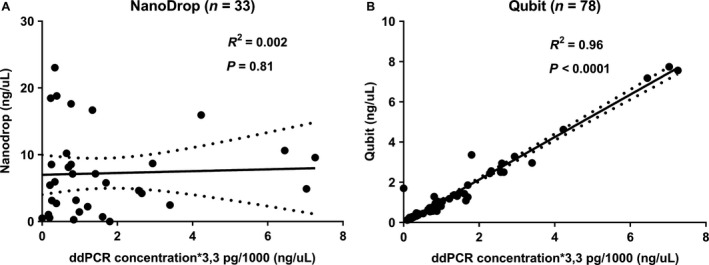
DNA quantification after isolation of EDTA samples using assay 3. In order to perform linear regression, all ddPCR results were adhered to NanoDrop and Qubit quantification results assuming 3.3 pg DNA/haploid genome (*x*‐axes) and depicted as ng/*μ*L (*y*‐axis). In total, 38 samples were quantified by NanoDrop (A), of which five results were negative values and excluded from analysis. Seventy‐eight samples were quantified using Qubit (B). *R* ^2^ represents goodness‐of‐fit of DNA quantification methods for ddPCR.

In order to assess accuracy of DNA quantification methods, results of NanoDrop and Qubit were compared to ddPCR results of identical plasma samples from healthy individuals. DNA quantification of samples using NanoDrop resulted in a nonsignificant *R* ^2^ for ddPCR using assay 3 (Fig. 6A), whereas Qubit results yielded *R* ^2^ of 0.96 (*β *= 1.06 [CI 1.01–1.10]), *P *<* *0.0001) for ddPCR (Fig. 6B). Results using assay 1 during ddPCR are shown in Fig. S5.

## Discussion

This study shows that ddPCR results of cfDNA quantification strongly depend on preanalytical blood sample workup, comprising an extensive multistep process. Our series of paired serum and plasma samples show a significant increase in median cfDNA concentrations of roughly 10 times in serum samples, which is consistent with several studies of comparable size reporting 2–24 times higher cfDNA concentrations detected by quantitative PCR in serum compared to plasma samples 12, 13, 14, 15. In our comparison of ddPCR results in various BCTs, no significant differences were found between ddPCR results of paired plasma and serum samples. This might have been the result of differences in sample size or workup. Furthermore, a significant increase in cfDNA concentrations has been previously observed in serum compared to plasma samples after storage time at room temperature and 4°C for up to 24 h 13. Possible explanations given for the increased DNA detection in serum samples are extracorporeal release of cfDNA from white blood cells (WBC) in serum through WBC lysis during whole blood transportation and centrifugation, or by stored clotted blood 16.

We assessed cfDNA concentrations in blood samples at different time points after isolation using two different methods. Our pooled data show that cfDNA concentrations can remain stable in EDTA plasma over 24 h until centrifugation. Furthermore, we found significantly stable cfDNA concentrations intraindividually, supported by ddPCR results of paired samples obtained at different time points. However, firm conclusions cannot be drawn due to small samples sizes. In one case, we observed an unexplained spike in cfDNA concentration at 3 h after ddPCR performed with two different assays. Previous studies showed no differences in cfDNA levels in plasma samples centrifuged at different time points up to 24 h 13, 16. On the contrary, several other studies reported significant increases in plasma cfDNA levels after storage of EDTA plasma up to 72 h at both room temperature and at 4°C to various degrees 7, 12, 17, 18. Differences in cfDNA yields among various studies might be the result of differences in sample handling and/or storage temperature, as this might affect WBC stability accompanied by the possible release of genomic DNA similar to serum samples 19.

Regarding cancer diagnostics, the detection of cfDNA mostly involves detecting mutations in ctDNA, which are often (rare) targets of interest within a background of wild‐type cfDNA 7. Therefore, variation in background DNA concentration during workup of serum samples is unwanted and should be avoided using plasma samples instead 20, 21. Furthermore, whole blood samples can be collected and stored for plasma analysis in BCTs containing different kinds of anticoagulants with potential ddPCR inhibiting features. Our results show that citrate and heparin anticoagulants yielded the smallest amounts of cfDNA from the same samples compared to serum and EDTA samples. This corresponds with previous qPCR results on different BCT types, where EDTA was preferred as anticoagulant above citrate or heparin as cfDNA concentrations appear to be more stable over time within EDTA matrix compared to citrate or heparin 22, 23. Palmirotta et al. compared DNA quality and quantity in blood plasma from healthy donors collected in six different BCTs containing different anticoagulants. Highest DNA purity and concentrations were reported for samples originating from citrate and EDTA plasma BCTs compared to heparin and fluoride‐oxalate BCTs, as measured by spectrophotometry, gel electrophoresis, and qPCR 24. More recently, newer commercially available BCTs specifically designed for cfDNA analysis are Cell‐Free DNA^™^ by Streck and CellSave by Janssen Diagnostics, which show even more stable plasma cfDNA concentrations after storage for 48 h up to 14 days compared to *K*_2_/*K*_3_‐EDTA BCTs 17, 25, 26, 27, 28, 29, 30. Therefore, the use of cfDNA and CellSave BCTs could be particularly beneficial for plasma cfDNA testing in large multicenter studies, in which storage time until centrifugation can practically be >24 h.

The centrifugation protocol for plasma collection also affects cfDNA concentration. Blood cells first have to be removed by slow centrifugation in order to avoid cell lysis and unwanted release of genomic DNA, whereas afterwards cellular remnants will be removed by short‐term high‐speed microcentrifugation, either before or after a freeze–thaw cycle 31. We observed a 2.5–3.0‐fold decrease in plasma cfDNA concentrations after a two‐step centrifugation compared to a single‐step slow‐speed centrifugation, which corresponds with previous data on protocols using similar centrifugation parameters and qPCR to quantify results 32, 33. We detected comparable results between two‐step protocols with modified forces and time parameters showing no significant differences in cfDNA yield, which is consistent with previous data on high‐speed centrifugation 33, 34. These results proof the (potentially unwanted) release of genomic DNA into the sample by remaining cellular material, and emphasizes the need for a second plasma filtering step by microcentrifugation, either pre‐ or post‐thaw, in order to prevent contamination with cellular DNA and retrieve purely plasma cfDNA.

Data on plasma storage conditions after centrifugation (e.g., time, temperature, freeze–thaw cycles) are scarce. Previously performed qPCR experiments showed comparable cfDNA yields for different parameters 7, 21. Furthermore, evidence exists that repeated freeze–thaw cycles of stored plasma samples prior to DNA isolation leads to cfDNA fragmentation 6, 7, 12.

Plasma DNA isolation can be performed using different methods supplied by a vast amount of manufacturers. Although sample sizes were small, we show that the five different DNA isolation kits we assessed performed all reasonably well; the Zymo Quick kit seemed to perform most efficiently in combination with ddPCR compared to the QIAamp and Jena PME kits, as highest absolute and relative concentrations of cfDNA were detected in plasma samples. On the other hand, the QIAamp kit showed the lowest coefficient of variation for both positive and total droplet yields, suggesting this kit to perform most consistently compared to the other two kits. Especially concerning cancer diagnostics (e.g., treatment response monitoring by serial quantification of ctDNA), consistency is an important factor to consider as only consistent quantification results would allow for reliable evaluation of tumor dynamics. Besides, the QIAamp kit revealed significantly higher droplet yields compared to the MagNA Pure kit. QIAamp and QIAsymphony performed equally well with regard to positive droplet yields. In a similar comparison with Jena PME and QIAsymphony, the QIAamp kit yielded highest concentrations of mutant KRAS in plasma samples from non‐small cell lung cancer patients 17. In two other studies, the Norgen Plasma/Serum Circulating DNA Purification Mini Kit yielded slightly better isolation results compared to the QIAamp kit depending on cancer type and used assay 9, while ctDNA in plasma samples of early stage (*KRAS*‐mutated) pancreatic cancer patients was not being detected using ddPCR after isolation with the QIAamp kit 35. Thus, isolation results not only vary strongly across different DNA isolation kits, but also between experiments performed with the same kit. Therefore, results not only depend on the used kit itself, but also on the circumstances and parameters used during experimental workup such as patient characteristics, tumor type and stage, target type, DNA input volume, and analysis technique. Overall, we experienced best ddPCR results of DNA isolation kits using Zymo Quick and QIAamp kits.

DNA isolation quantity could be checked by fluorospectroscopy or fluorometry of DNA eluates. *R* ^2^ demonstrated a poor predictive ability of NanoDrop quantification measurements in respect to ddPCR cfDNA concentrations of eluted DNA samples, while a strong significant correlation was found between Qubit quantification measurements and ddPCR results of these samples. Similar results were previously shown for experiments using qPCR 9. We observed no correlation between NanoDrop measurements and ddPCR results. Therefore, we recommend a DNA isolation quantification check using Qubit fluorometry before proceeding to actual PCR analysis. This could enhance efficiency during workflow by avoiding wasting of time and costly materials used during ddPCR, in case of insufficient DNA isolated.

Several variables during preparation of actual ddPCR can affect analysis results. For instance, the amount of DNA sample input to be analyzed, which largely depends on the purpose of testing; maximum available amounts of DNA sample are desired in case of rare target detection (e.g., cancer diagnostics, post‐transplantation monitoring), while for copy number variation analysis (e.g. prenatal diagnostics) the amount of required DNA depends on the expected highest target copy number. Assays used for ddPCR need to be validated separately, because fluorescence values used for readout of positive and negative droplets can vary depending on PCR inhibitors present in DNA matrix or assay design. First, optimal ddPCR settings (i.e., ramp‐rate and annealing time) should be determined by temperature gradients performed on positive control samples with similar DNA matrix. Subsequently, the limit of detection (LOD) needs to be determined by estimating false‐positive rate through running strings of wild‐type‐only control and NTC samples 36. During post‐PCR analysis, technical errors such as reduced or increased fluorescent signals from damaged positive droplets or negative droplets can cause droplets to be displayed in between positive or negative clusters, which is defined as “rain”. This hampers accurate post‐PCR analysis. Third parties already designed methods to improve automated thresholding by either *k*‐nearest neighbor clustering, “extreme value methodology”, or kernel density estimation with Gaussian mixture models 37, 38, 39. Lastly, the interpretation of results remains subject of discussion. Positive targets in Quantasoft are being reported as copies/*μ*L reaction volume, which is calculated using the number of droplets detected and droplet volume (nano sized) 40. Subsequently, this could manually be converted to copies/mL plasma and whole blood using input volume of DNA sample for ddPCR and plasma volume for DNA isolation. However, large conversion factors between these volumes could easily render errors in estimating target copy concentrations in blood. Therefore, standardization of input volumes for analysis is pivotal. This further raises important questions about the clinical relevance of the acquired results; what is the significance of a decrease or increase of target concentrations in clinical management? At what target concentration should (targeted) therapy be started and/or adjusted?

In conclusion, we recommend a two‐step centrifugation protocol for separating plasma collected in EDTA BCTs for storage until cfDNA isolation within 24 h. Furthermore, the Zymo Quick kit yielded best results quantitatively for cfDNA isolation compared to others. The QIAamp kit seems to be most consistent and yielded highest cfDNA concentrations compared to the QIAsymphony and MagNA Pure kits. Furthermore, we think that Qubit fluorometry for a quantity check of cfDNA isolation might enhance workflow efficiency towards ddPCR analysis. And, although further clinical research and technical refinements of ddPCR analysis are needed for incorporation into clinical practice, improving overall efficiency in sample workup is an inevitable first step.

## Conflict of Interest

All authors state that there were no conflicts of interest.

## Supporting information


**Figure S1.** The boxplots indicate cfDNA concentrations as shown on the y‐axis, while comparing citrate, heparin, serum, and EDTA BCTs from 8 healthy controls as shown on the x‐axis.

**Figure S2.** Influence of storage time until centrifugation on cfDNA concentrations in paired EDTA samples from 6 healthy individuals after PCR using assay 3.

**Figure S3.** Additional comparison of centrifugation protocols A‐C in EDTA samples from D12‐D17 show similar results using assay 3, validating the results of this experiment using assay 2: median cfDNA concentrations detected after centrifugation using protocol A were 77.5 [21.6–166.3] copies/*µ*L, compared to 27.1 [13.6–39.6] copies/µL using protocol B and 30.8 (13.3–114.5) copies/µL using protocol C.

**Figure S4.** Isolation methods in healthy individuals using assay 4. Healthy individuals (D43‐D46) are depicted on the x‐axes.

**Figure S5.** DNA quantification of EDTA samples prior to ddPCR using assay 1. Forty‐four samples were quantified using both methods.

**Table S1.** Summary of material and methods used during workup experiments.
**Table S2.** Overview of ddPCR assays following MIQE guidelines.
