# Supplementary figures and images for: Preanalytical blood sample workup for cell‐free DNA analysis using Droplet Digital PCR for future molecular cancer diagnostics
Source: Cancer Med. 2017 Sep 21;6(10):2297–307. doi: 10.1002/cam4.1184 (PMC5633557; doi:10.1002/cam4.1184)

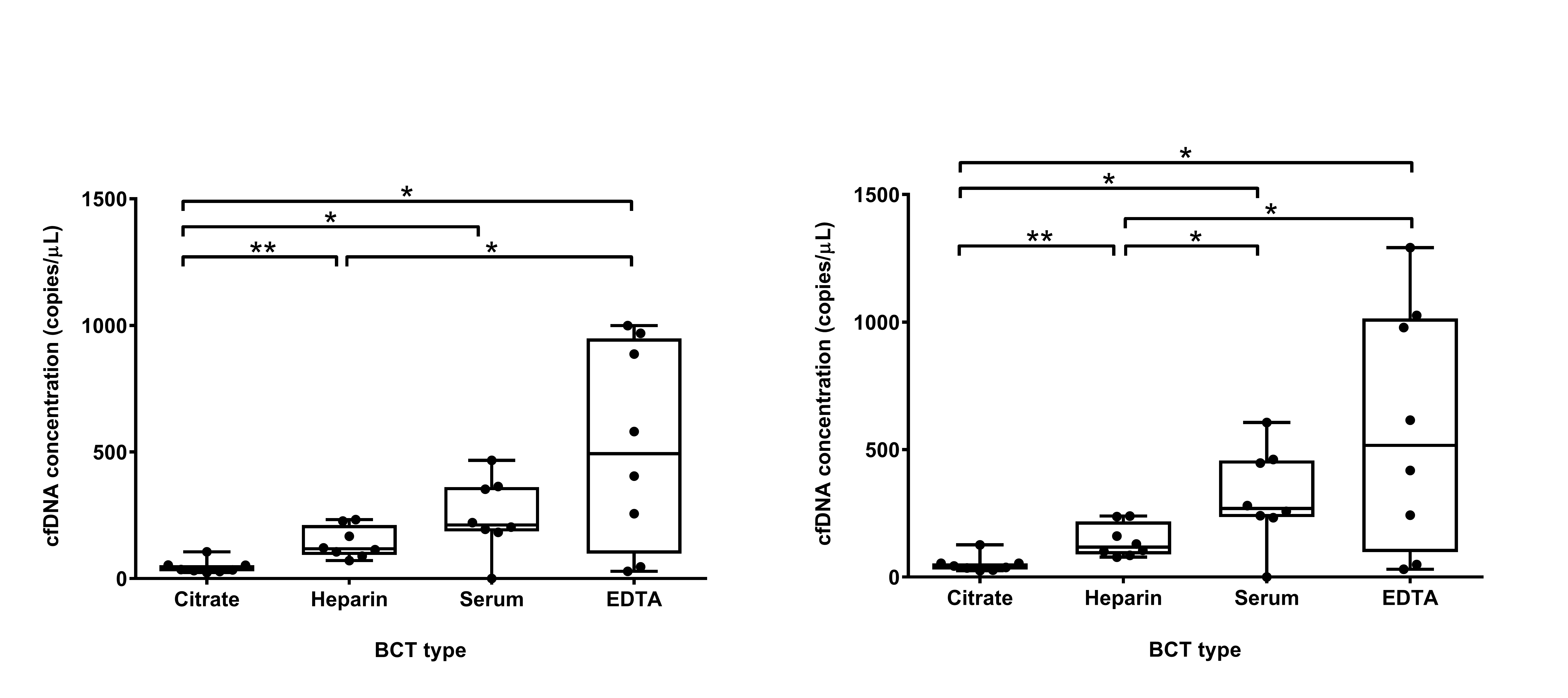

Supplement: Supplementary file 1 — Figure S1. The boxplots indicate cfDNA concentrations as shown on the y‐axis, while comparing citrate, heparin, serum, and EDTA BCTs from 8 healthy controls as shown on the x‐axis. [file CAM4-6-2297-s001.tif]

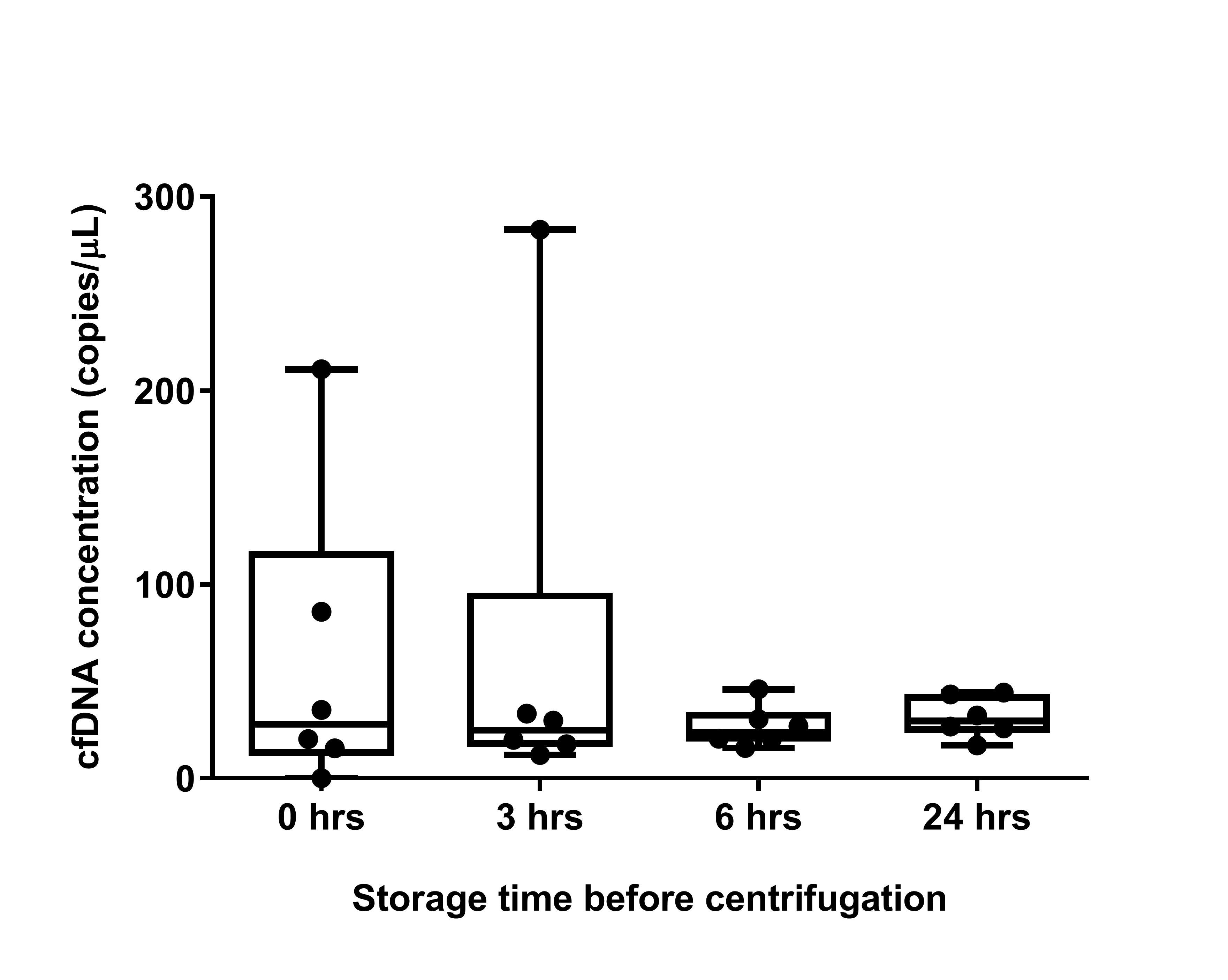

Supplement: Supplementary file 2 — Figure S2. Influence of storage time until centrifugation on cfDNA concentrations in paired EDTA samples from 6 healthy individuals after PCR using assay 3. [file CAM4-6-2297-s002.tif]

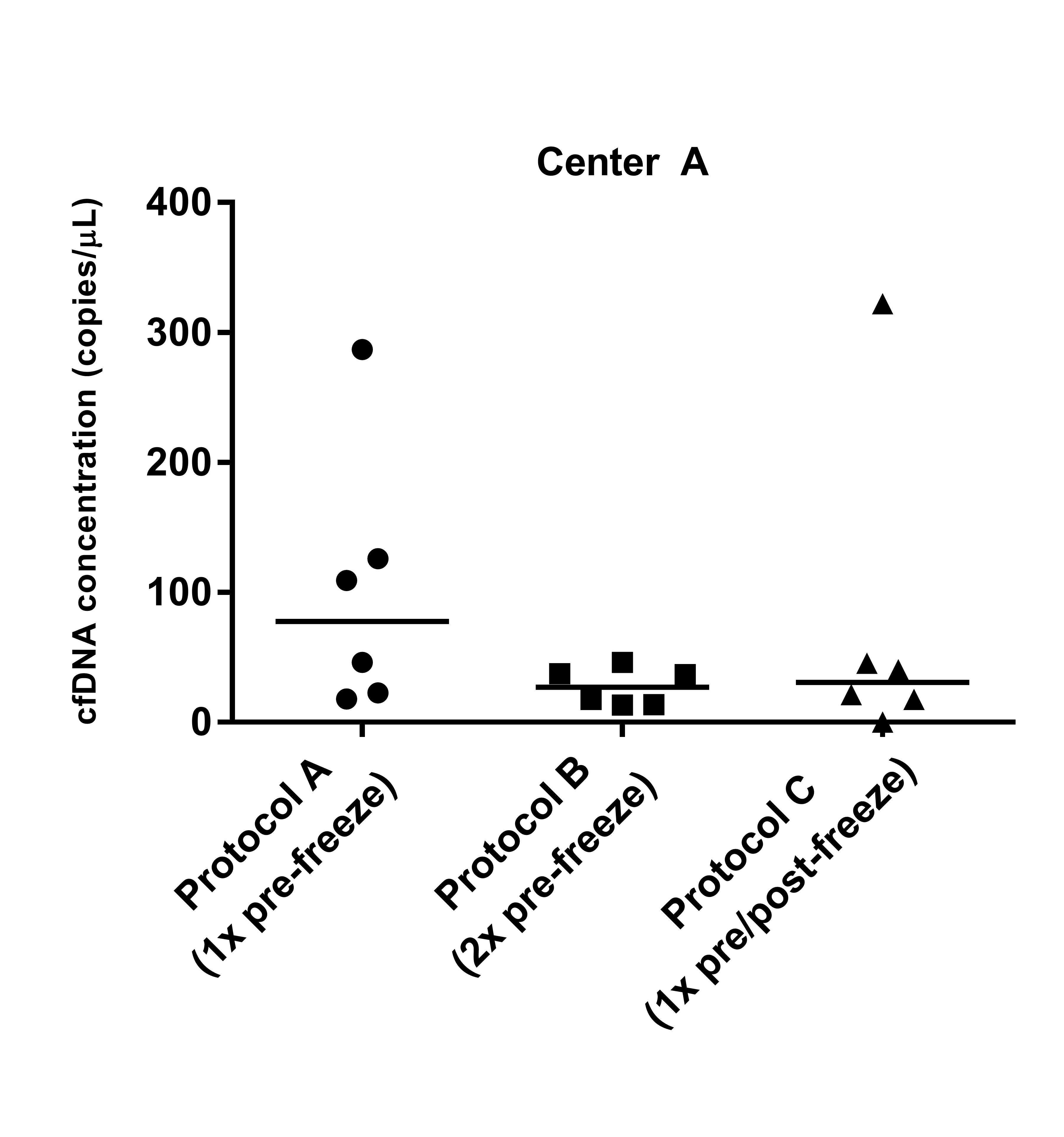

Supplement: Supplementary file 3 — Figure S3. Additional comparison of centrifugation protocols A‐C in EDTA samples from D12‐D17 show similar results using assay 3, validating the results of this experiment using assay 2: median cfDNA concentrations detected after centrifugation using protocol A were 77.5 [21.6–166.3] copies/µL, compared to 27.1 [13.6–39.6] copies/µL using protocol B and 30.8 (13.3–114.5) copies/µL using protocol C. [file CAM4-6-2297-s003.tif]

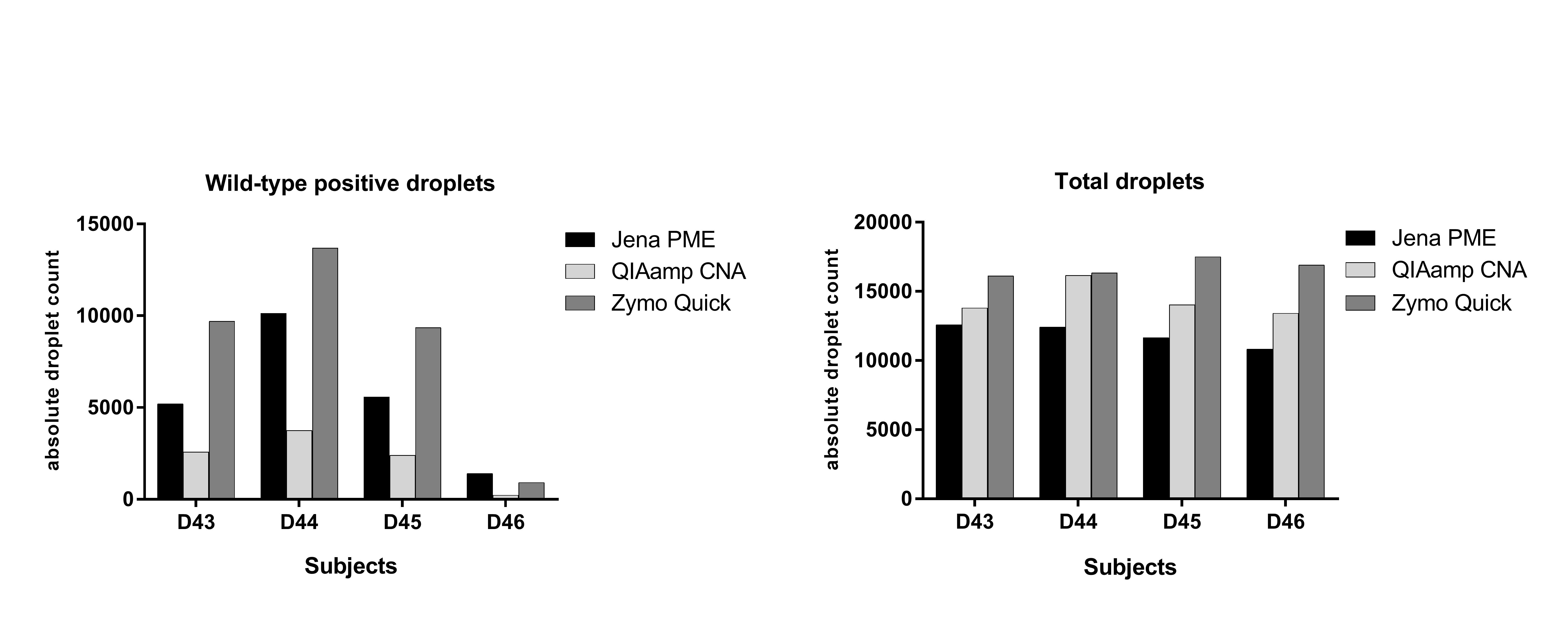

Supplement: Supplementary file 4 — Figure S4. Isolation methods in healthy individuals using assay 4. Healthy individuals (D43‐D46) are depicted on the x‐axes. [file CAM4-6-2297-s004.tif]

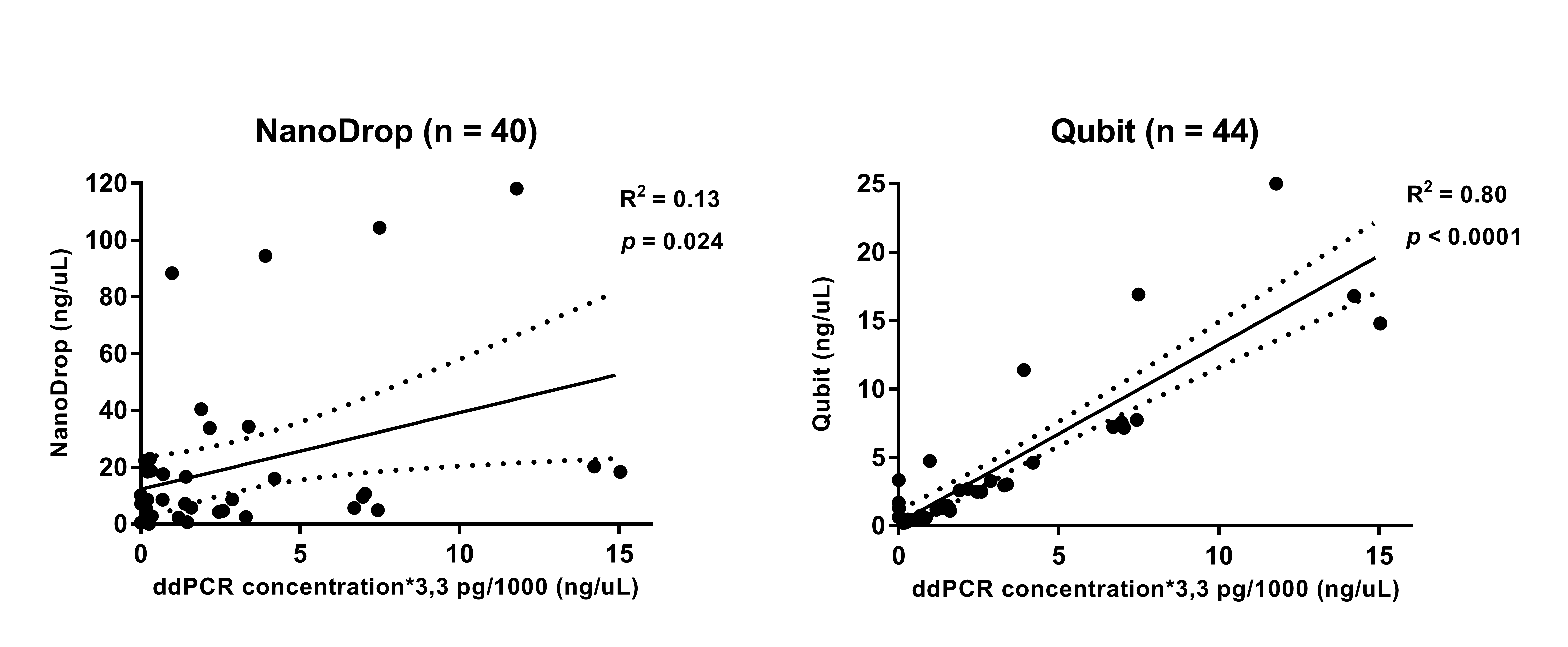

Supplement: Supplementary file 5 — Figure S5. DNA quantification of EDTA samples prior to ddPCR using assay 1. Forty‐four samples were quantified using both methods. [file CAM4-6-2297-s005.tif]
