# Supplementary material for: Preanalytical blood sample workup for cell‐free DNA analysis using Droplet Digital PCR for future molecular cancer diagnostics
Source: Cancer Med. 2017 Sep 21;6(10):2297–307. doi: 10.1002/cam4.1184 (PMC5633557; doi:10.1002/cam4.1184)
Supplement: Supplementary file 6 — Table S1. Summary of material and methods used during workup experiments. Table S2. Overview of ddPCR assays following MIQE guidelines. [file CAM4-6-2297-s006.docx]

| **Subject** | **Patient type** | **Applied method** | | | | |
| --- | --- | --- | --- | --- | --- | --- |
|  |  | **BCT^1^** | **Storage time (T)²** | **Centrifugation³** | **DNA isolation^4^** | **Assay^5^** |
| D1 | Healthy donor | E, S | 1 | D | M | 1 |
| D2 | Healthy donor | E, S | 1 | D | M | 1 |
| D3 | Healthy donor | E, S | 1 | D | M | 1 |
| D4 | Healthy donor | E, S | 1 | D | M | 1 |
| D5 | Healthy donor | E, S | 1 | D | M | 1 |
| D6 | Healthy donor | E, S | 1 | D | M | 1 |
| D7 | Healthy donor | E, S | 1 | D | M | 1 |
| D8 | Healthy donor | E, S | 1 | D | M | 1 |
| D9 | Healthy donor | E, S | 1 | D | M | 1 |
| D10 | Healthy donor | E, S | 1 | D | M | 1 |
| D11 | Healthy donor | E, S | 1 | D | QA | 1 |
| D12 | Healthy donor | E, S | 1 | D | QA | 1 |
| D13 | Healthy donor | E, S | 1 | D | QA | 1 |
| D14 | Healthy donor | E, S | 1 | D | QA | 1 |
| D15 | Healthy donor | E, S | 1 | D | QA | 1 |
| D16 | Healthy donor | E, S | 1 | D | QA | 1 |
| D17 | Healthy donor | E, S | 1 | D | QA | 1 |
| D18 | Healthy donor | E, S | 1 | D | QA | 1 |
| D19 | Healthy donor | E, S | 1 | D | QA | 1 |
| D20 | Healthy donor | E, S | 1 | D | QA | 1 |
| D21 | Healthy donor | E, S | 1 | D | QA | 1 |
| D22 | Healthy donor | E, S | 1 | D | QA | 1 |
| D23 | Healthy donor | E, S | 1 | D | QA | 1 |
| D24 | Healthy donor | E, S | 1 | D | QA | 1 |
| D25 | Healthy donor | E, S | 1 | D | QA | 1 |
| D26 | Healthy donor | E, H, S, C | 1 | A | Z | 1,2,3 |
| D27 | Healthy donor | E, H, S, C | 1 | A | Z | 1,2,3 |
| D28 | Healthy donor | E, H, S, C | 1 | A | Z | 1,2,3 |
| D29 | Healthy donor | E, H, S, C | 1 | A | Z | 1,2,3 |
| D30 | Healthy donor | E, H, S, C | 1 | A | Z | 1,2,3 |
| D31 | Healthy donor | E, H, S, C | 1 | A | Z | 1,2,3 |
| D32 | Healthy donor | E, H, S, C | 1 | A | Z | 1,2,3 |
| D33 | Healthy donor | E, H, S, C | 1 | A | Z | 1,2,3 |
| D34 | Healthy donor | E | 1,2,3,4 | A | Z | 2,3 |
| D35 | Healthy donor | E | 1,2,3,4 | A | Z | 2,3 |
| D36 | Healthy donor | E | 1,2,3,4 | A | Z | 2,3 |
| D37 | Healthy donor | E | 1,2,3,4 | A,B,C | Z | 2,3 |
| D38 | Healthy donor | E | 1,2,3,4 | A,B,C | Z | 2,3 |
| D39 | Healthy donor | E | 1,2,3,4 | A,B,C | Z | 2,3 |
| D40 | Healthy donor | E | 2 | A,B,C | Z | 2,3 |
| D41 | Healthy donor | E | 2 | A,B,C | Z | 2,3 |
| D42 | Healthy donor | E | 2 | A,B,C | Z | 2,3 |
| D43 | Healthy donor | E | 2 | A | J, Z, QA | 1,4 |
| D44 | Healthy donor | E | 2 | A | J, Z, QA | 1,4 |
| D45 | Healthy donor | E | 2 | A | J, Z, QA | 1,4 |
| D46 | Healthy donor | E | 2 | A | J, Z, QA | 1,4 |
| P1 | NSCLC | E, S | 2 | D, E | QS | 1 |
| P2 | NSCLC | E, S | 2 | D, E | QS | 1 |
| P3 | NSCLC | E, S | 2 | D, E | QS | 1 |
| P4 | NSCLC | E, S | 2 | D, E | QS | 1 |
| P5 | NSCLC | E, S | 2 | D, E | QS | 1 |
| P6 | NSCLC | E | 2 | D | M, QA, QS | 5 |
| P7 | NSCLC | E | 2 | D | M, QA, QS | 6 |
| P8 | NSCLC | E | 2 | D | M, QA, QS | 6 |
| P9 | NSCLC | E | 2 | D | M, QA, QS | 5 |
| P10 | NSCLC | E | 2 | D | M, QA, QS | 7 |
| P11 | NSCLC | E | 2 | D | M, QA, QS | 8 |
| P12 | NSCLC | E | 2 | D | M, QA, QS | 6 |
| P13 | NSCLC | E, St, CS | 1,4 | D | M | 9 |
| P14 | NSCLC | E, St, CS | 1,4 | D | M | 11 |
| P15 | NSCLC | E, St, CS | 1,5 | D | M | 10 |
| P16 | NSCLC | E, St, CS | 1,5 | D | M | 5 |
| P17 | NSCLC | E, St, CS | 1,6 | D | M | 6 |
| P18 | NSCLC | E, St, CS | 1,6 | D | M | 11 |
| Pool 1 | Various cancers | E | 1,2,3,4 | D | M, QS | 1 |
| Pool 2 | Various cancers | E | 1,2,3,4 | D | M, QS | 1 |
| Pool 3 | Various cancers | E | 1,2,3,4 | D | M, QS | 1 |

**Supplementary Table S1.** Summary of material and methods used during workup experiments

^1^ E EDTA; S, Serum; H, Heparin; C, Citrate; St, Streck BCT; CS, CellSave BCT.

^2^ 1 = direct; 2 = 3 h; 3 = 6 h; 4 = 24 h; 5 = 48 h; 6 = 5 days.

^3^ A, 1x pre-freeze; B, 2x pre-freeze; C, 1x pre-freeze/1x post-thaw; D, 2x pre-freeze; E, 1x pre-freeze/1x post-thaw.

^4^ J, Jena PME; QA, QIAamp; QS, QIAsymphony; M, MagNA Pure; Z, Zymo Quick.

^5^ 1, BRAF_intron_; 2, RRP30_intron_; 3, EIF2C1_intron_; 4, LEPREL2_intron_; 5, EGFR_c.2236G>C_; 6, EGFR_c.2235_2249del15_; 7, EGFR_c.2369C>T_; 8, EGFR_c.2155G>A_; 9, EGFR_c.2582T>A_; 10, KRAS_c.34G>T_; 11, KRAS_multplex_

| Assay | Supplier | ID | Gene | Chromosome mapping | Chromosome location | variant | Protein change | Amplicon Context [FAM/HEX] | Amplicon length |
| --- | --- | --- | --- | --- | --- | --- | --- | --- | --- |
| 1 | Bio-Rad | dHsaCP2000028 | BRAF | 7q34 | 7:140500162-40500284 | Intron | N/A | CACTCCATCGAGATTTCACTGTAGCTAGACCAAAATCACCTATTTTTACTGTGAGGTCTTCATGAAGAAATATATCTGAGGTGTAGTAAGTAAAGGAAAACAGTAGATCTCATTTTCCTATCA | 91 |
| 2 | Bio-Rad | dHsaCP1000485 | RRP30 | 10q23.31 | 10:92634445-92634567 | Intron | N/A | TTAAGTAACTTGTAAGTGGTAGTGCATAGACTTTAAATCAGGCAGACTGACACTAGAGTTCACATTCATAACCACTCCTCAAATGTCCTCCTACTCTTGACATCTAGACTCAGGATGGACCTG | 98 |
| 3 | Bio-Rad | dHsaCP1000484 | EIF2C1 | 1p34.3 | 1:36359357-36359479 | Intron | N/A | GGCTTTCACCAGTCTGTGCGCCCTGCCATGTGGAAGATGATGCTCAACATTGATGGTGAGTGGGGAGAGCTATGGAGCCAGGGGCACCCCAAGTCCAGTGACCACACTCCCAGCCTCATCCCT | 86 |
| 4 | Life technologies | rs2269355 | LEPREL2 | 12q13 | 12:6836700-6836800 | Intron | N/A | TGTAGGAAGCTCTCCCGAGTTCTCT[G/C]CACAGTCCCCTTAGTAAGCGGGATT | 51 |
| 5 | Bio-Rad | dHsaCP2000021 | EGFR | 7p12 | 7:55259483-55259605 | c.2236G>C | L858R | ACCGCAGCATGTCAAGATCACAGATTTTGGGCTGGCCAAACTGCTGGGTGCGGAAGAGAAAGAATACCATGCAGAAGGAGGCAAAGTAAGGAGGTGGCTTTAGGTCAGCCAGCATTTTCCTGA | 73 |
| 6 | Bio-Rad | dHsaCP2000039 | EGFR | 7p12 | 7:55242438-55242560 | c.2235_2249del15 | E746-A750del | GAAAGTTAAAATTCCCGTCGCTATCAAGGAATTAAGAGAAGCAACATCTCCGAAAGCCAACAAGGAAATCCTCGATGTGAGTTTCTGCTTTGCTGTGTGGGGGTCCATGGCTCTGAACCTCAG | 78 |
| 7 | Bio-Rad | dHsaCP2000019 | EGFR | 7p12 | 7:55249042-55249164 | c.2369C>T | T790M | CTGCCTCACCTCCACCGTGCAGCTCATCACGCAGCTCATGCCCTTCGGCTGCCTCCTGGACTATGTCCGGGAACACAAAGACAATATTGGCTCCCAGTACCTGCTCAACTGGTGTGTGCAGAT | 80 |
| 8 | Bio-Rad | dHsaIS2504192 | EGFR | 7p12 | 7:55241679-55241801 | c.2155G>A | G719S | AACTGAATTCAAAAAGATCAAAGTGCTGGGCTCCGGTGCGTTCGGCACGGTGTATAAGGTAAGGTCCCTGGCACAGGCCTCTGGGCTGGGCCGCAGGGCCTCTCATGGTCTGGTGGGGAGCCC | 65 |
| 9 | Bio-Rad | dHsaCP2000043 | EGFR | 7p12 | 7:55259493-55259615 | c.2582T>A | L861Q | GTCAAGATCACAGATTTTGGGCTGGCCAAACTGCTGGGTGCGGAAGAGAAAGAATACCATGCAGAAGGAGGCAAAGTAAGGAGGTGGCTTTAGGTCAGCCAGCATTTTCCTGACACCAGGGAC | 64 |
| 10 | Bio-Rad | dHsaMDV2510584 | KRAS | 12p12.1 | 12:25398224-25398346 | c.34G>T | G12C | ATTATTTTTATTATAAGGCCTGCTGAAAATGACTGAATATAAACTTGTGGTAGTTGGAGCT[G/T]GTGGCGTAGGCAAGAGTGCCTTGACGATACAGCTAATTCAGAATCATTTTGTGGACGAATA | 57 |

**Supplementary Table S2.1.** Overview of ddPCR assays following MIQE guidelines

**Supplementary Table S2.2.** KRAS G12/G13 Screening Multiplex Assay

| **Assay** | **Supplier** | **Gene** | **Chromosome mapping** | **ID** | **Chromosome location** | **variant** | **Protein change** | **Amplicon Context [FAM/HEX]** | **Amplicon length** |
| --- | --- | --- | --- | --- | --- | --- | --- | --- | --- |
| **11** | Bio-Rad | KRAS | 12p12.1 | dHsaMDV2510586 | 12:25398223-25398345 | c.35G>C | G12A | TTATTTTTATTATAAGGCCTGCTGAAAATGACTGAATATAAACTTGTGGTAGTTGGAGCTG[G/C]TGGCGTAGGCAAGAGTGCCTTGACGATACAGCTAATTCAGAATCATTTTGTGGACGAATAT | 57 |
|  |  |  |  | dHsaMDV2510584 | 12:25398224-25398346 | c.34G>T | G12C | ATTATTTTTATTATAAGGCCTGCTGAAAATGACTGAATATAAACTTGTGGTAGTTGGAGCT[G/T]GTGGCGTAGGCAAGAGTGCCTTGACGATACAGCTAATTCAGAATCATTTTGTGGACGAATA | 57 |
|  |  |  |  | dHsaMDV2510596 | 12:25398223-25398345 | c.35G>A | G12D | TTATTTTTATTATAAGGCCTGCTGAAAATGACTGAATATAAACTTGTGGTAGTTGGAGCTG[G/A]TGGCGTAGGCAAGAGTGCCTTGACGATACAGCTAATTCAGAATCATTTTGTGGACGAATAT | 57 |
|  |  |  |  | dHsaMDV2510590 | 12:25398224-25398346 | c.34G>C | G12R | ATTATTTTTATTATAAGGCCTGCTGAAAATGACTGAATATAAACTTGTGGTAGTTGGAGCT[G/C]GTGGCGTAGGCAAGAGTGCCTTGACGATACAGCTAATTCAGAATCATTTTGTGGACGAATA | 57 |
|  |  |  |  | dHsaMDV2510588 | 12:25398224-25398346 | c.34G>A | G12S | ATTATTTTTATTATAAGGCCTGCTGAAAATGACTGAATATAAACTTGTGGTAGTTGGAGCT[G/A]GTGGCGTAGGCAAGAGTGCCTTGACGATACAGCTAATTCAGAATCATTTTGTGGACGAATA | 57 |
|  |  |  |  | dHsaMDV2510592 | 12:25398223-25398345 | c.35G>T | G12V | TTATTTTTATTATAAGGCCTGCTGAAAATGACTGAATATAAACTTGTGGTAGTTGGAGCTG[G/T]TGGCGTAGGCAAGAGTGCCTTGACGATACAGCTAATTCAGAATCATTTTGTGGACGAATAT | 57 |
|  |  |  |  | dHsaMDV2510598 | 12:25398220-25398342 | c.38G>A | G13D | TTTTTATTATAAGGCCTGCTGAAAATGACTGAATATAAACTTGTGGTAGTTGGAGCTGGTG[G/A]CGTAGGCAAGAGTGCCTTGACGATACAGCTAATTCAGAATCATTTTGTGGACGAATATGAT | 57 |

**Supplementary Figure S1.** The boxplots indicate cfDNA concentrations as shown on the y-axis, while comparing citrate, heparin, serum, and EDTA BCTs from 8 healthy controls as shown on the x-axis. The crossing lines indicate medians, the upper and lower limits of the boxes indicate interquartile ranges (25^th^/75^th^ percentiles), and whiskers represent minima and maxima. Using assay 2 (A), significantly higher cfDNA concentrations were found in EDTA samples compared to paired heparin samples and citrate samples (493.0 [98.7 – 948.5] vs. 117.5 [93.3 – 212.0] and 35.1 [28.5 – 53.3], both *p* < 0.05). Compared to citrate samples, median cfDNA concentrations in both paired serum and heparin samples were significantly higher (212.0 [186.0 – 361.3] and 117.5 [93.3 – 212.0] vs. 35.1 [28.5 – 53.3], *p <*0.01 and *p <*0.05). Using assay 3 (B), significantly higher cfDNA concentrations were found in EDTA samples compared to paired heparin and citrate samples (516.5 [97.7 – 1014.0] vs. 117.5 [89.0 – 218.0] and 40.6 [29.4 – 54.0], both *p <*0.05). Furthermore, serum samples contained significantly higher cfDNA concentrations compared to paired heparin and citrate samples (269.0 [235.0 – 457.5] vs. 117.5 [89.0 – 218.0] and 40.6 [29.4 – 54.0], both *p <*0.05), as well as heparin samples contained significantly higher cfDNA concentrations compared to citrate samples (117.5 [89.0 – 218.0] and 40.6 [29.4 – 54.0], *p* < 0.01). *P < 0.05, **P < 0.01, ***P < 0.001).

**Supplementary Figure S2.** Influence of storage time until centrifugation on cfDNA concentrations in paired EDTA samples from 6 healthy individuals after PCR using assay 3. Time points T_1_-T_4_ are shown on the x-axes and median cfDNA concentrations on the y-axes. No significant differences were found between median DNA concentrations at consecutive time points T_1_-T_4_.

**Supplementary Figure S3.** Additional comparison of centrifugation protocols A-C in EDTA samples from D12-D17 show similar results using assay 3, validating the results of this experiment using assay 2: median cfDNA concentrations detected after centrifugation using protocol A were 77.5 [21.6 – 166.3] copies/µl, compared to 27.1 [13.6 – 39.6] copies/µl using protocol B and 30.8 (13.3 – 114.5) copies/µl using protocol C.

**Supplementary Figure S4.** Isolation methods in healthy individuals using assay 4. Healthy individuals (D43-D46) are depicted on the x-axes. Absolute droplet counts are shown on the y-axes for both wild-type (A) and total positive droplet (B). The gray scaled bars represent 3 different commercially available isolation kits.

**Supplementary Figure S5.** DNA quantification of EDTA samples prior to ddPCR using assay 1. Forty-four samples were quantified using both methods. After excluding 4 quantification results from analysis due to negative values, NanoDrop resulted in R^2^ of 0.13 (β= 2.87[CI 0.65 - 5.10], *p* < 0.05). For Qubit, R^2^ was 0.80 (β = 1.30[CI 1.10 - 1.50]), *p* < 0.0001) for ddPCR.
